# Clinical Presentation of COVID-19 and Antibody Responses in Bangladeshi Patients Infected with the Delta or Omicron Variants of SARS-CoV-2

**DOI:** 10.3390/vaccines10111959

**Published:** 2022-11-18

**Authors:** Asish Kumar Ghosh, Olfert Landt, Mahmuda Yeasmin, Mohiuddin Sharif, Rifat Hossain Ratul, Maruf Ahmed Molla, Tasnim Nafisa, Mymuna Binte Mosaddeque, Nur Hosen, Md. Rakibul Hassan Bulbul, Rashid Mamunur, Alimul Islam, Shahjahan Siddike Shakil, Marco Kaiser, Md. Robed Amin, Simon D. Lytton

**Affiliations:** 1 Department of Virology, Dhaka Medical College Hospital, Dhaka 1000, Bangladesh; 2 TIB Molbiol GmbH, Eresburgstraße 22-23, 12103 Berlin, Germany; 3 National Institute of Laboratory Medicine and Referral Center, Sher E-Bangla Nagar, Dhaka 1207, Bangladesh; 4 Department of Medicine, Dhaka Medical College Hospital, Dhaka 1000, Bangladesh; 5 Department of Virology, Bangabandhu Sheikh Mujib Medical University, Shahbag, Dhaka 1000, Bangladesh; 6 Institute for Developing Science & Health Initiatives, Dhaka 1216, Bangladesh; 7 Bangladesh Institute Tropical Infectious Disease (BITID), Fouzderhat, Chittagong 4317, Bangladesh; 8 Department of Microbiology and Hygiene, Faculty of Veterinary Science, Bangladesh Agricultural University, Mymensingh 2202, Bangladesh; 9 National Institute of Diseases of the Chest and Hospital, Mohakhali, Dhaka 1212, Bangladesh; 10 SeraDiaLogistics, 81545 Munich, Germany

**Keywords:** SARS CoV-2 variants, Omicron, Delta, PCR melting curve analysis, COVID-19 symptoms, hospitalization, anti-N protein, anti-S protein

## Abstract

The clinical presentation of COVID-19 and the specific antibody responses associated with SARS-CoV-2 variants have not been investigated during the emergence of Omicron variants in Bangladesh. The Delta and Omicron variants were identified by post-PCR melting curve analysis of the spike (S) protein receptor binding domain amplicons. Anti-S-protein immunoglobulin-G anti-nucleocapsid (N)-protein immunoglobulin-G and immunoglobulin-A levels were measured by ELISA. The Delta variant was found in 40 out of 40 (100%) SARS-CoV-2 RT-PCR positive COVID-19 patients between 13 September and 23 October 2021 and Omicron variants in 90 out of 90 (100%) RT-PCR positive COVID-19 patients between 9 January and 10 February 2022. The Delta variant associated with hospitalization (74%, 80%, and 40%) and oxygen support (60%, 57%, and 40%) in the no vaccine, dose-1, and dose-2 vaccinated cases, respectively, whereas the Omicron COVID-19 required neither hospitalization nor oxygen support (0%, *p* < 0.0001). Fever, cough, and breathlessness were found at a significantly higher frequency among the Delta than Omicron variants (*p* < 0.001). The viral RNA levels of the Delta variant were higher than that of the Omicron variants (Ct median 19.9 versus 23.85; *p* < 0.02). Anti-spike protein immunoglobulin-G and anti-N-protein immunoglobulin-G within 1 week post onset of Delta variant COVID-19 symptoms indicate prior SARS-CoV-2 infection. The Delta variant and Omicron BA.1 and BA.2 breakthrough infections in the Dhaka region, at 240 days post onset of COVID-19 symptoms, negatively correlated with the time interval between the second vaccine dose and serum sampling. The findings of lower anti-spike protein immunoglobulin-G reactivity after booster vaccination than after the second vaccine dose suggest that the booster vaccine is not necessarily beneficial in young Bangladeshi adults having a history of repeated SARS-CoV-2 infections.

## 1. Introduction

The severe acute respiratory syndrome coronavirus 2 (SARS-CoV-2) Wuhan Hu-1 strain, the original causative agent of the coronavirus disease 2019 (COVID-19) pandemic, has undergone a high frequency of mutations in the spike-(S) protein [1]. The variants are assigned Greek alphabetical letters by the WHO [2] and cross-referenced to the clade nomenclature of international reference network laboratories GISAID, Nextstrain, and Pango [3]. The “variants of concern” (VOCs) are classified in accordance with the criteria of increased transmission fitness, virulence, and/or immune evasion [2,4]. Meta-analysis of twenty six independent clinical studies between June 2020 and October 2021 on COVID-19 outcomes indicate that the Beta and Delta VOCs were associated with greater than 2-fold increased risk of COVID-19 related hospitalization, ICU admission, and mortality compared with the original wild type SARS CoV-2 [5]. The first retrospective observational studies in South Africa and Denmark reported a lower risk of hospitalization with Omicron infection compared with Delta infection among both vaccinated and non-vaccinated individuals [6,7]. Prospective observational study in the United Kingdom reported lower prevalence in the loss of smell, less involvement of the lower respiratory tract, and a shorter period of illness in Omicron variants compared with Delta variant infections [8]. The clinical manifestations and epidemiological aspects of COVID-19 have been extensively reported in various regions around the world [9]. The SARS CoV-2 Omicron variants in Bangladesh have not been evaluated with respect to the COVID-19 outcomes.

The deletion at S-protein amino acids 69–70, recognized as a signature mutation of the Omicron variant, was used to detect the Omicron variant [10]. The BA.2 Omicron variant lacks this deletion, and is thus termed the “stealth variant” because its genetic mutations were not distinguished from the Delta variant using standard PCR testing. New methodologies involving post-PCR melting curve analysis were subsequently developed for the detection of Omicron variants [11,12,13].

The charged lysine and arginine residues at positions N440K, T478K, Q493R, N501Y, and Q498R in the RBD of Omicron variants have been implicated in the increased angiotensin converting enzyme-2 (ACE-2) receptor binding affinity relative to the Delta variant and Omicron’s immune evasion from vaccine-induced neutralizing antibodies [14]. In September 2021, one year and six months after Bangladesh’s nation-wide lockdown, the PCR positive SARS CoV-2 declined to approximately two percent [15] and the reproductive rate to Rt of less than one [16]. In January 2022, the Bangladesh Institute of Epidemiology Disease Control and Research (IEDCR) confirmed the identification of the Omicron BA.1 and BA.2 variants in 118 out of 148 (80%) of SARS CoV-2 RT-PCR swab samples at which time the RT-PCR positivity rate of 30 percent was reported [17,18]. SARS-CoV-2 Omicron infection peaked on 7 January 2022 in the United Kingdom while the Omicron wave in Bangladesh began on 5 January 2022 and reached a peak of 86 RT-PCR positive confirmed COVID-19 cases per million on 29 January 2022 [15,19]. Although anecdotal evidence suggests that the Omicron variants were less pathogenic than other SARS-CoV-2 VOCs, there was uncertainty at this time regarding the micron COVID-19 severity among non-European ethnic and demographic groups [20,21,22]. The reports on mucomycosis associated with the COVID-19 Delta variant in India [23,24], the coronavirus disease-associated pulmonary aspergillosis [25], and the evidence that Omicron variants evade neutralizing antibodies produced by previous coronavirus infection or vaccination [26] were alarming to the Bangladeshi population that was facing insufficient vaccine dosages and limited oxygen supply [22,27].

To follow-up on our previous molecular surveillance of the SARS CoV-2 Delta variant in Bangladesh in 2021 [28], the current study focused on the COVID-19 symptoms and hospitalization in Dhaka at a period of transition—the end of the infection wave with the SARS CoV-2 Delta variant and the emergence of Omicron variants.

## 2. Materials and Methods

### 2.1. Study Design and Sample Collections

This study was a cross-sectional single-center study on two cohorts of Bangladeshi adults testing RT-PCR SARS-CoV-2 positive Group 1: forty with symptomatic COVID-19 between 23 September and 13 October 2021; twenty eight with no vaccine, and twelve with either one or two doses of AstraZeneca vaccine. Group 2 included ninety with either no symptoms or mild COVID-19 symptoms between 9 January 2022 and 10 February 2022 at Dhaka Medical College; eighty four received two doses of AstraZeneca COVISHIELD vaccine and six had no vaccine administration. The data analysis in this study was limited by the small sample size of Group 1 vaccination (Delta) and the small sample size of Group 2 with no vaccine (Omicron). All COVID-19 cases in the two groups were randomly sampled for the survey of SARS CoV-2 variants. Nasopharyngeal specimens were collected and stored at −80 °C in a virus collection and preservation medium (Khang Jian Medical Apparatus Ltd., Taizhou, China) and transported on dry ice to Germany. Sera matched to the date of swab collection cohort 1, n = 50, and at follow-up on day 19 to day 27, n = 25, post onset of COVID-19 symptoms (POCS) were kept at −20 °C storage in screw-tight cryopreservation vials (Greiner-bio one).

### 2.2. Quantification of Viral RNA

The viral RNA of nasopharyngeal specimens was extracted with a Magnapure 96 instrument (Roche, Mannheim, Germany) using the DNA and Viral Nucleic Acid Kit (Roche, Penzberg, Germany). The RNA concentration in each sample was determined by reverse transcriptase polymerase chain reaction (RT-PCR) using the LightMix^®^ Modular SARS-CE assay (40-0770 and 60-0770, TIB MolBiol, Berlin, Germany) and programming on a 480II light cycler (Roche, Penzberg, Germany). The point at which the amplification curve of the E gene crossed the vertical threshold line in the RT PCR cycle (Ct) was reported as positive if the sample Ct was less than 40.

### 2.3. The Spike Protein Variants

H69V70del, Y144del, T478K, K417N/T, P681R/H, E484A, and N501Y were identified using a RT-PCR melting curve analysis targeting amino acid mutations. The VirSNiP SARS-CoV-2 typing assays (TibMolBiol, Cat. No. 53-0781-96, 53-0799-96, 53-0807-96, 53-0811-96, 53-0813-96) and LightCycler^®^ Multiplex RNA Virus Master (Roche Cat. No. 06 754 155 001) were performed following the manufacturer’s instructions.

### 2.4. Assessment of Anti-SARS CoV-2 Antibodies

The positive anti-nucleocapsid (N)-protein IgA and IgG reactivity were according to the recommended cut-off value of NovaTec units (NTU) >10 for positive results (Novatec Diagnostics, Dietzenbach, Germany); NTU = X * 10/QC, where X = OD_450nm_ − OD_620nm_ of the test sample and QC = OD_450nm_ − OD_620nm_ of the quality control equivocal serum sample. E > 10 NTU. The anti-SARS-CoV-2 spike (S) protein IgG QuantiVac ELISA (EUROIMMUN Medizinische Labordiagnostika AG, Germany) was measured according to the manufacturer’s instructions. The values of OD_450nm_ − OD_620nm_ were reported at serial dilutions of serum. All ELISA measurements were performed on a Multiskan 96-well reader (Lot 357-706872, Thermofischer Scientific Life Technologies, Darmstadt, Germany).

### 2.5. Statistical Analysis

The Ct values, the levels of the anti-SARS CoV-2 S-proteins IgA and IgG, and the levels of the anti-N-protein IgG are presented as the mean with a standard deviation and as medians with ranges. Comparisons of the values between patient groups were assessed by a nonparametric Mann–Whitney sum rank test or Chi-square analysis. A *p* value of ≤0.05 was considered statistically significant with differences between independent groups. Correlation analyses were calculated with the Spearman’s rank correlation coefficients. The correlation coefficients r > 0.4 or r < −0.4 with significance at *p* < 0.05 were considered strong positive or strong negative associations, respectively. Statistical analysis was performed with MedCalc version 14 for Windows (MedCalc Software, Mariakerke, Belgium).

## 3. Results

### 3.1. Bangladeshi COVID-19 Patient Characteristics

Two groups of COVID-19 patients were investigated. Table 1 shows the number and percent of hospitalization, oxygen support, and symptoms in the COVID-19 cases with versus without vaccination. The differences in the proportions between the two groups were of significance with respect to hospitalization, oxygen support, and symptoms. In Group 1, exclusively the Delta variant, 40 percent hospitalization and 40 percent oxygen support were observed in the vaccinated versus 70 percent and 64 percent, respectively, in the non-vaccinated. In Group 2, all 90 cases were Omicron infections without hospitalization or the need for oxygen support. No co-infections of the Delta variant and Omicron variants were identified.

### 3.2. SARS CoV-2 Variants

**Figure 1:**
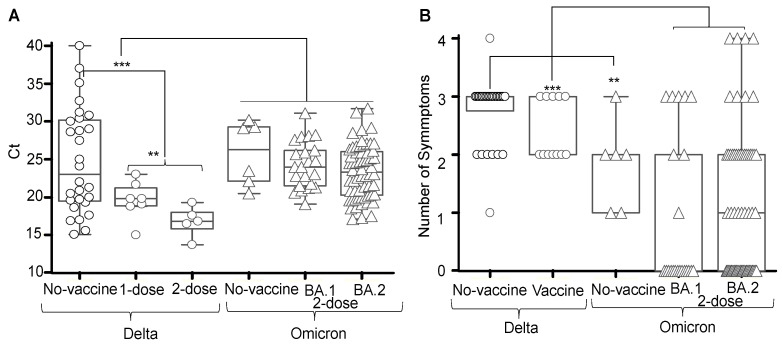
Viral loads and symptoms in the COVID-19 Delta variant versus the COVID-19 Omicron variants. Viral loads (**A**) and Symptoms (**B**). ** *p* < 0.02 and *** *p* < 0.0001. Delta variant Ct median 19.9 (95% CI 19.3–22.8 and micron variants Ct median 23.85 (95% CI 22.8–24.8).

The viral loads of the Delta variant were higher than the viral loads of the Omicron variants, as indicated by the significantly lower median Ct of the Delta variant than the median Ct of the micron variants (Figure 1A).

The number of symptoms in COVID-19 with the Delta variant were significantly greater than the number of symptoms in COVID-19 with Omicron variants (Figure 1B). Neither the number of symptoms nor the type of symptoms correlated with the viral loads (results not shown).

### 3.3. Antibody Responses in SARS-CoV-2 Variants

Sera from the COVID-19 Group 1 of the Delta variant but not the Group 2 of the Omicron variants were available for the evaluation of antibody responses during the hospitalization period and after COVID-19 recovery (Table 2).

The anti-SARS CoV-2 S-protein IgG and anti-N-protein IgG and anti-N protein IgA were measured in forty patients on day of RT-PCR within 7 days post onset of COVID-19 symptoms (POCS) and in twenty five patients at follow-up sera sampling on day 19 to day 27 of POCS. Table 2 shows that both the vaccinated and non-vaccinated patients were probably infected previously with another SARS CoV-2 lineage, since 100 percent of the samples exhibited anti-N-protein IgG.

On day 1 to day 7 POCS, the anti-N protein IgA positivity in the non-vaccinated COVID-19 cases and the COVID-19 cases that received one dose of vaccine was 82% and 86%, respectively, was higher than the anti-N protein IgA positivity in COVID-19 cases that received two doses of vaccine; 40%.

**Figure 2:**
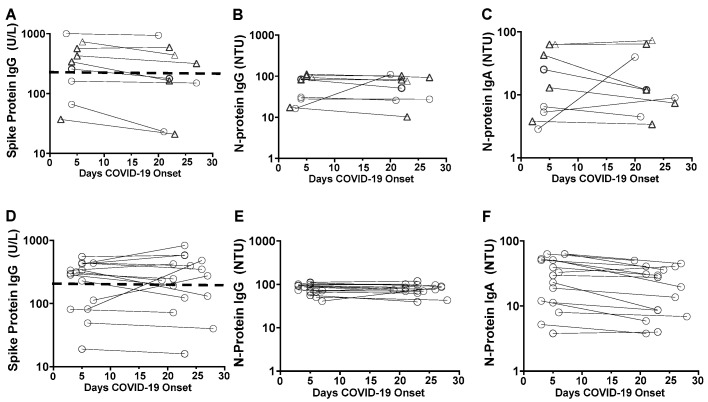
Prospective monitoring of antibody levels in the vaccinated (**A**–**C**) and non-vaccinated COVID-19 patients (**D**–**F**). The horizontal dotted line of 250 NTU represents the antibody activity saturation level at 1:100 serum dilution.

At follow-up on day 19 to day 27 POCS, 25 out of 25 tested (100%) gave positive anti-S protein IgG and 24 out of the 25 sera tested (96%) gave positive anti-N protein IgG positivity. With the exception of spike protein IgG reactivity in one vaccinated patient and two patients without vaccination and the anti-N protein reactivity of one vaccinated patient, no significant changes were found in the IgG levels between the time of swab sampling and the follow-up time points (Figure 2). Anti-S protein IgG activity above the saturation level at both time points in the Novalisa Kit was shown in six out of nine (67%) in the vaccinated cases versus 12 out of 16 (75%) in the cases without vaccination.

**Figure 3:**
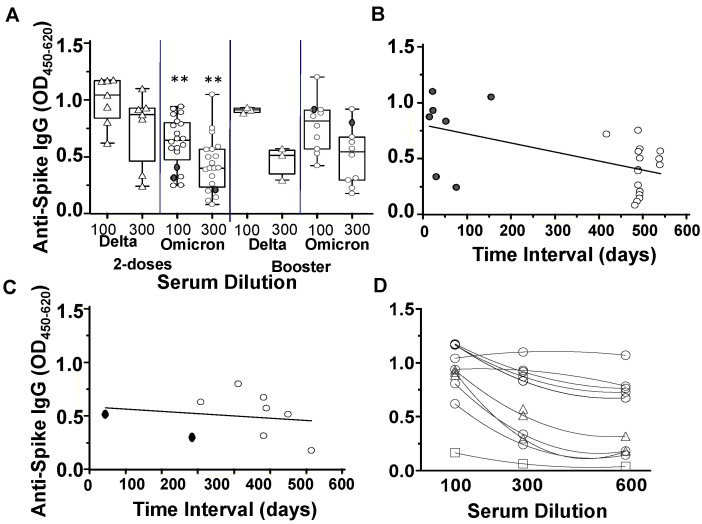
Anti-spike protein IgG reactivity 210 to 240 days POCS follow-up. Horizontal axis is either the serum dilutions (**A**,**D**) or the time intervals between serum sampling and last vaccination (**B**,**C**) The Delta variant n = 11 (triangles) versus the Omicron variant n = 30 (circles). Three Omicron cases reported a second natural infection 60 to 180 days prior to the serum sampling, bold circle (**A**). Linear regression of the anti-S-protein IgG reactivity, OD_450nm_ − OD_620nm_ at 1:300 serum dilution, from the Delta variant (closed circles) or Omicron variant infections (open circles) versus the time interval between the second vaccine dose and serum sampling; r = −0.31 *p* < 0.01 (**B**) or time interval between the booster vaccine and serum sampling; r = −0.03, *p* = 0.64 (**C**). Titers of anti-spike protein IgG in delta variant infections, n = 10; 1-dose (open rectangle), 2-doses (open circles) and booster (open triangles). Serum diluted 100-fold, 300-fold and 600-fold in Euroimmun Quant ELISA (**D**). ** *p* < 0.001 independent student *t*-test differences of the mean OD_450nm_ − OD_620nm_ IgG values Delta variant mean of 0.94 (95% CI 0.8–1.09) and mean 0.75 (95% CI 0.45–1.05) versus the Omicron variants’ mean of 0.65 (95% CI 0.57–0.74) and mean 0.43 (95% CI 0.32–0.54) at 1:100 and 1:300 dilutions, respectively.

At 210 to 240 days POCS, ten cases of the Delta variant infection and thirty cases of the Omicron variant infections were assessed for anti-S-protein IgG reactivity. At this follow-up time interval, the Delta variant cases had received a second vaccine dose within 30 to 150 days and the Omicron variant cases received a second vaccine dose between 450 and 550 days prior to the serum sampling. In the cases receiving two vaccine doses, the anti-S-protein IgG of the Delta variant showed significantly greater reactivity than the anti-S-protein IgG reactivity of the Omicron variants (Figure 3A) and negatively correlated with the time interval between the second vaccine dose and the date of serum sampling (Figure 3B). In the cases receiving the vaccine booster, the IgG reactivity between the Delta versus Omicron infection groups was of no significance (Figure 3A,C). The anti-spike protein IgG levels of the Omicron booster were slightly higher than that of the Omicron 2-dose, but the differences were of no significance (Figure 3A). A second breakthrough infection was reported in three out of 30 (10%) Omicron and 0 out of 11 (0%) Delta variant infections 90 to 180 days prior to the serum sampling. Two of the second breakthrough infections occurred 30 to 60 days prior to the second vaccine dose and showed anti-S-protein IgG reactivity in the lowest IQR (Figure 3A). The titers of the anti-S-protein IgG at 210 to 240 days POCS of Delta variant infection were determined by serial serum dilutions and found to be consistently higher among the seven cases receiving two vaccine doses than among the three cases receiving the booster or one case of one vaccine dose (Figure 3D).

**Figure 4:**
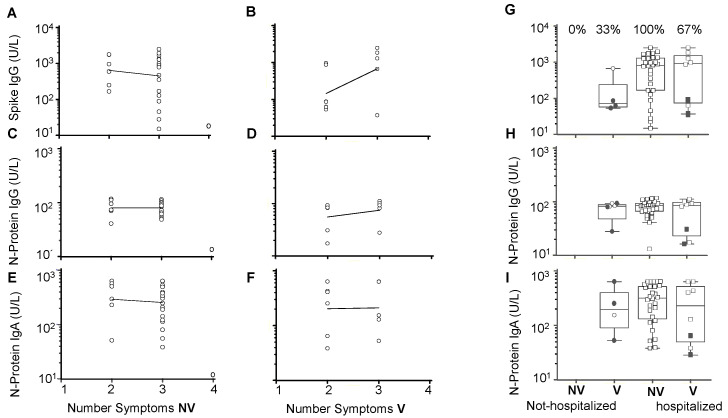
Antibody responses in SARS CoV-2 Delta variant infection and associations with COVID-19 symptoms and hospitalization. Linear regression analysis of the anti-S protein IgG, the anti-N protein IgG, and the anti-N protein IgA levels versus the number of symptoms in non-vaccinated COVID-19 (**A**,**C**,**E**); coefficients of correlation r = −0.04 (95% CI −0.41–0.34), *p* = 0.884; r = −0.06 (95% CI −0.43–0.33), *p* = 0.78; r = −0.095 (95% CI −0.46–0.3), *p* = 0.6, respectively. Anti-S protein IgG, (**B**), anti-N protein IgG (**D**), and N-protein IgA (**F**) versus the number of symptoms in one or two dose vaccinated COVID-19; r = 0.5 (95% CI −0.17–0.833), *p* = 0.1, r = 0.24 (95% CI −0.416–0.74), *p* = 0.47 and r = 0.02 (95% CI −0.58–0.6), *p* = 0.95, respectively. The distribution of anti-S protein IgG (**G**), anti-N protein IgG (**H**), and anti-N protein IgA (**I**) levels in non-hospitalized versus hospitalized COVID-19. NV = not vaccinated, V = vaccinated. Filled symbols represent cases receiving two doses of vaccine. The percentages (%) of V and NV are indicated.

To assess whether or not the antibody responses of the Delta variant infection on day 19 to day 27 POCS are associated with symptoms, linear regression analyses were performed between the number of symptoms versus the levels of anti-S protein IgG, anti-N-protein IgG, or anti-N protein IgA. The single non-vaccinated patient who reported four symptoms and low antibody reactivity was excluded from the regression analysis (Figure 4).

The antibody levels negatively correlated with the number of COVID-19 symptoms in the non-vaccinated Delta variant infections without significance (Figure 4A,C,E). In the vaccinated COVID-19 Delta variant, the anti-S protein IgG levels showed a trend to positively correlate with the number of symptoms (Figure 4D) but without significance. No associations were found between the anti-N protein IgG levels or the anti-N-protein IgA levels versus the number of symptoms (Figure 4E,F).

Figure 4G shows the anti-spike protein IgG levels in the six non-hospitalized cases of the COVID-19 Delta variant significantly lower than the levels in the 34 hospitalized cases. In contrast, neither the anti-N-protein IgG levels nor the anti-N protein IgA levels showed significant differences between the hospitalized versus non-hospitalized COVID-19 Delta variant (Figure 4H,I).

## 4. Discussion

The expansion of the SARS CoV-2 Omicron variants into Bangladesh at the beginning of 2022 was associated with COVID-19 having ostensibly mild symptoms and no hospitalization. Omicron variants have been reported worldwide and have largely replaced the BA.1 and BA.2 variants which are associated with mild febrile illness and significant reduction in the frequency of lower respiratory tract symptoms [29]. At the time of this study, the BA.1 and BA.2 Omicron variants had not been systematically documented in Bangladeshis of different ages and gender with or without vaccination.

The trend of higher RNA levels among recipients of the second vaccine dose than RNA levels of no vaccine reinforces the evidence that commercial vaccines do not disrupt SARS CoV-2 transmission but are rather favorable for the spread of SARS CoV-2 variants [30,31]. An alternative conclusion is that the commercial vaccine in Bangladesh allowed individuals to better resist against active viral infection. The vaccinated cases that eventually fell sick with COVID-19 only did so as they were exposed to, and subsequently infected by, a much higher viral load than that which would be needed to actively infect a non-vaccinated individual. This interpretation is partially supported by the apparent relationship that non-vaccinated individuals showing more symptoms (and so sicker) despite having a lower viral load than vaccinated individuals (Figure 1). The rapid replacement of the Delta variant by Omicron variants in the Dhaka region occurred approximately one month following the first Omicron wave in the United Kingdom, and previous to this study, the Omicron variant infections in Bangladesh were cited only in news reports and government bulletins in December 2021.

The central weakness of this study was the small sample size, five completely vaccinated COVID-19 Delta variant in Group 1, and the age bias in the six non-vaccinated COVID-19 Omicron cases sampled in Group 2. Another limitation was that no data were available on antibody responses at the intervals of day 1 to 7 and days 19 to 27 in the Group 2 COVID-19 patients with Omicron variant infections.

The high levels of anti-N-protein IgG found within five days POCS of the Delta variant infection indicate prior exposure to coronaviruses. We acknowledge that a large proportion of the Delta variant infections had anti-spike protein IgG activity reaching saturation in the Novalisa Kit, which may obscure changes at the follow-up time point. The trend for the number of symptoms to negatively correlate with the anti-S protein IgG in the Delta variant infections without vaccination suggest that high titers of neutralizing antibodies are induced by Delta variant natural infection and are beneficial for the recovery from COVID-19. Although the rates of COVID-19 related hospitalization and oxygen support were lower in the vaccinated Delta variant infections compared to the Delta variant infections without vaccination, the number of symptoms showed a trend to positively associate with anti-S-protein IgG levels in breakthrough Delta infection. This finding, although biased due to the small sample size, suggests that the levels of neutralizing antibodies during Delta variant breakthrough infection increase in accordance with the acute symptomatic antiviral response whereas the vaccine-induced adaptive immunity rather than titers of spike neutralizing antibodies per se are critical for the prevention of severe COVID-19 disease [32,33]. To what extent antibodies generated during repeated natural infections interfere with the activity of vaccine-induced neutralizing antibodies should be cautiously interpreted due to the small sample size of COVID-19 illness in twice vaccinated Bangladeshis.

The lower anti-spike protein IgG reactivity found in the Omicron variant infections compared to the anti-spike protein IgG reactivity in the Delta variant infections at day 210 to day 240 POCS were of strong statistical significance and likely attributed to the waning of antibody titers beyond 6 months post-vaccination. In the absence of reference data on ELISA reactivity at day 9 to day 27 POCS for the Omicron variant infections, we cannot rule out the role of immune evasion on neutralizing antibody responses [34]. Breakthrough infections and/or administration of refresher or booster vaccine typically increases the neutralization potency and durability of anti-S protein IgG levels [35,36]. The findings of anti-spike protein IgG reactivity consistently lower in both the Omicron and Delta variant receiving a booster vaccine compared with anti-spike protein IgG reactivity after the second vaccine dose suggest that the booster vaccine did not necessarily increase the titers of neutralizing antibodies. On this basis, we recommend that health authorities carefully consider the history of repeated SARS CoV-2 exposure and the severity of Omicron breakthrough infections before administering the vaccine booster to young adults in Bangladesh.

## 5. Conclusions

The random sampling of nasopharyngeal specimens from two independent COVID-19 groups in the Dhaka region during two successive and non-overlapping periods revealed how the predominance of the Delta variant in September and October 2021 was abruptly replaced by Omicron variants in January and February 2022.

The distinct clinical outcomes in these two Bangladeshi COVID-19 cohorts underscore how the unique properties of the VOCs affect COVID-19 severity. The post RT-PCR melting curve analyses is an effective and practical tool to identify SARS CoV2 variants. For this reason, we recommend its implementation in a low income country such as Bangladesh, which has well-established nationwide facilities and training on SARS CoV-2 PCR testing albeit insufficient resources for performing routine genomic sequencing.

Molecular surveillance of VOCs by PCR and measurement of the antibody levels by ELISA are practical and essential tools in responsible public health decisions. The increase in SARS-CoV-2 infections with mild illness and the complacency among fully vaccinated populations to disregard social distancing and the wearing of facial masks has led to the perception that “the COVID-19 pandemic is over”. This likely misconception must not deter efforts to continue the monitoring and identification of SARS CoV-2 variants and the pursuit of pandemic preparedness.

## Figures and Tables

**Table 1:** Patient Characteristics. The patient characteristics were compared in two groups of SARS-CoV RT-PCR positive COVID-19 in the Dhaka region: Group 1 in the period between 13 September and 23 October 2021, and Group 2 in the period between 9 January and 10 February 2022. The *p*-value *p* (1) represents the difference between no vaccine Group 1 versus no vaccine group 2. The *p*-value *p* (2) represents the difference between Group 1 receiving either one or two vaccine doses versus Group 2 receiving two vaccine doses. The differences between the median age, the proportion of gender, and the median days post onset of COVID-19 symptoms (POCS) were determined by the Mann–Whitney sum rank test. The differences between the number of cases n and (percent) between the two groups were determined by Chi-square analysis. * Omicron mixed refers to the detection of both the BA.1and BA.2 sub-variants in the same patient swab sample.

Characteristics	SARS-CoV-2 RT-PCR Positive 2021 Group 1			SARS-CoV-2 RT-PCR Positive 2022 Group 2		*p* Value (1)	*p* Value (2)
	No Vaccine	1-dose	2-dose	No Vaccine	2-dose		
**Number of cases enrolled**	28	7	5	6	84		
**Age Median (range) years**	50 (23–85)	50 (24–62)	50 (27–68)	12 (7–95)	37 (19–66)	0.002	0.09
**Gender M/F**	11/17	4/3	5/0	2/4	73/11	<0.0001	0.5
Days POCS median (range)	5 (1–7)	4 (2–6)	4 (3–6)	3 (1–4)	2 (1–4)	0.0001	0.0001
Hospitalized n (%)	28 (100)	6 (86)	2 (40)	0 (0)	0 (0)	0.0001	0.0001
Difficulty breathing n (%)	23 (82)	4 (57)	2 (40)	0 (0)	10 (12)	0.0001	0.001
Oxygen support n (%)	23 (82)	4 (57)	2 (40)	0 (0)	0 (0)	0.0001	0.0001
**Variant n (%)**							
Delta	28 (100)	7 (100)	5 (100)	0 (0)	0 (0)	0.0001	0.0001
Omicron BA.1	0 (0)	0 (0)	0 (0)	1 (17)	22 (26)		
Omicron BA.2	0 (0)	0 (0)	0 (0)	5 (83)	60 (72)		
* Omicron mixed	0 (0)	0 (0)	0 (0)	0 (0)	2 (2)		
**COVID-19 symptoms n (%)**							
No symptoms	0 (0)	0 (0)	0 (0)	0 (0)	38 (45)	0.0001	0.008
Rhinitis	0 (0)	0 (0)	0 (0)	4 (67)	27 (32)	0.02	0.05
Chest pain	0 (0)	2 (3)	0 (0)	0 (0)	0 (0)	0.9	0.9
Breathlessness	22 (79)	4 (6)	2 (40)	0 (0)	8 (10)	0.0001	0.001
Muscle or body pain or headache	1 (3)	1 (1.5)	3 (60)	1 (17)	12 (14)	0.25	0.21
Nausea	1 (3)	0 (0)	0 (0)	0 (0)	0 (0)	0.56	0.99
Loss of smell and/or taste	4 (14)	0 (0)	0 (0)	0 (0)	0 (0)	0.02	0.99
Cough	24 (86)	6 (86)	1 (20)	4 (67)	12 (14)	0.017	0.001
Fever	27 (96)	5 (71)	5 (100)	3 (50)	32 (38)	0.0001	0.009

**Table 2:** Anti-SARS CoV-2 antibody responses during the Delta variant infection.

Antibody Positivity Days Post Onset of COVID-19 Symptoms (POCS)	SARS-CoV-2 RT-PCR Positive 2021 Group 1			
	Non- Vaccinated	1-dose Vaccine	2-dose Vaccine	*p* Value
SARS-CoV-2 NP IgA n (%)				
Day 1–7	23 (82)	6 (86)	2 (40)	0.12
Day 19–27	10 (62)	5 (60)	2 (50)	0.98
SARS-CoV-2 NP IgG n (%)				
Day 1–7	28 (100)	7 (100)	5 (100)	-
Day 19–27	16 (100)	5 (80)	4 (100)	-
SARS-CoV-2 S IgG Quant n (%)				
Day 1–7	28 (100)	7 (100)	5 (100)	-
Day 19–27	16 (100)	5 (80)	4 (100)	-

## Data Availability

All of the sequencing data and information in this study is available in GISAID. Accession numbers are provided.
